# Manually curated genome-scale reconstruction of the metabolic network of *Bacillus megaterium* DSM319

**DOI:** 10.1038/s41598-019-55041-w

**Published:** 2019-12-10

**Authors:** Javad Aminian-Dehkordi, Seyyed Mohammad Mousavi, Arezou Jafari, Ivan Mijakovic, Sayed-Amir Marashi

**Affiliations:** 1 Biotechnology Group, Department of Chemical Engineering, Tarbiat Modares University, Tehran, Iran; 2 Department of Chemical Engineering, Tarbiat Modares University, Tehran, Iran; 3 Department of Biology and Biological Engineering, Chalmers University of Technology, Göteborg, Sweden; 4 Novo Nordisk Foundation Center for Biosustainability, Technical University of Denmark, Lyngby, Denmark; 5 Department of Biotechnology, College of Science, University of Tehran, Tehran, Iran

**Keywords:** Metabolic engineering, Bacterial systems biology, Metabolic engineering, Applied microbiology

## Abstract

*Bacillus megaterium* is a microorganism widely used in industrial biotechnology for production of enzymes and recombinant proteins, as well as in bioleaching processes. Precise understanding of its metabolism is essential for designing engineering strategies to further optimize *B*. *megaterium* for biotechnology applications. Here, we present a genome-scale metabolic model for *B*. *megaterium* DSM319, *i*JA1121, which is a result of a metabolic network reconciliation process. The model includes 1709 reactions, 1349 metabolites, and 1121 genes. Based on multiple-genome alignments and available genome-scale metabolic models for other *Bacillus* species, we constructed a draft network using an automated approach followed by manual curation. The refinements were performed using a gap-filling process. Constraint-based modeling was used to scrutinize network features. Phenotyping assays were performed in order to validate the growth behavior of the model using different substrates. To verify the model accuracy, experimental data reported in the literature (growth behavior patterns, metabolite production capabilities, metabolic flux analysis using ^13^C glucose and formaldehyde inhibitory effect) were confronted with model predictions. This indicated a very good agreement between *in silico* results and experimental data. For example, our *in silico* study of fatty acid biosynthesis and lipid accumulation in *B*. *megaterium* highlighted the importance of adopting appropriate carbon sources for fermentation purposes. We conclude that the genome-scale metabolic model *i*JA1121 represents a useful tool for systems analysis and furthers our understanding of the metabolism of *B*. *megaterium*.

## Introduction

In recent decades, research on *Bacillus megaterium* has gained momentum due to its versatile metabolic capabilities and physical properties favorable to biotechnology applications. This bacterium had already been commonly used in biochemical studies before the extensive popularity of *Bacillus subtilis*^1,2^. Large cell size and special physiochemical properties were the main incentives to use *B*. *megaterium* as a model to study cell structure, sporulation, and protein localization^3,4^. As a Gram-positive bacterium with an aerobic sporulation behavior, *B*. *megaterium* inhabits diverse environments, ranging from dried food to soil^5^. Its ability to grow on a variety of carbon sources has made it amenable for industrial applications^6^. Numerous strains of *B*. *megaterium* have been applied for production of various enzymes, such as penicillin amidase, amylase, amino acid dehydrogenase, and glucose dehydrogenase, as well as for production of recombinant proteins^7–11^. Moreover, it has been used as an alternative microorganism for production of vitamin B_12_, pyruvate, and shikimate^12–14^. Remarkably, *B*. *megaterium* can also be utilized as a cyanogenic bacterium in the bioleaching process, in order to mobilize precious metals from e-wastes^15^.

Among various strains of *B*. *megaterium*, *B*. *megaterium* DSM319 (*B*. *m*. DSM319 hereafter) stands out as being extensively investigated. *B*. *m*. DSM319 and its available derivatives are commonly used for production of recombinant proteins^16–18^ and metabolites^19–22^. The whole genome of *B*. *m*. DSM319 was sequenced by Eppinger *et al*.^23^, which provided a wealth of information on its genotype-phenotype relationships. However, genome data alone was not sufficient for providing a holistic and comprehensive picture of the *B*. *m*. DSM319 metabolism. Therefore, there remains a knowledge gap that needs to be filled.

Genome-scale metabolic networks together with constraint-based modeling provide a computational framework that can predict physiological features of cells and organisms^24^. Genome-scale metabolic models (GEMs) are used to evaluate and determine the potential of industrial strains and explore their unknown metabolic capabilities^25^. GEM-based predictions can be used for amelioration of culture medium composition, by identifying key metabolites that need to be added to increase the growth rate. They can also be used for predicting metabolite production fluxes^26^ and defining gene deletion strategies for metabolic engineering^27^.

**Figure 1:**
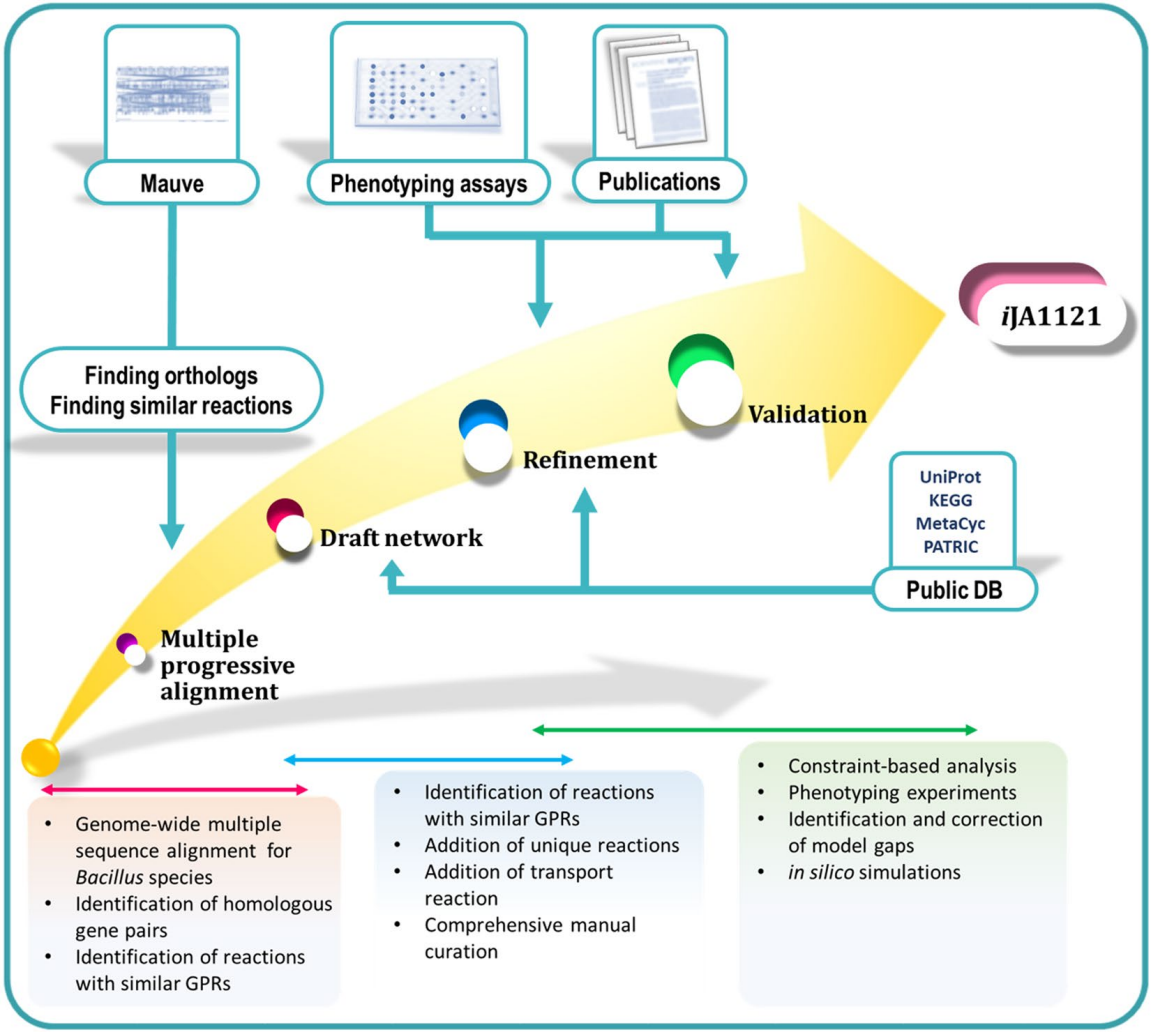
Schematic representation of the genome-scale metabolic network reconstruction procedure. Using Mauve, homologous gene pairs were detected to identify the reactions with similar gene-reaction associations in genome-scale metabolic models of *Bacillus* species. After the comprehensive manual curation, the draft model was validated and refined using phenotyping experiments and the experimental data reported in the literature.

A GEM for *B*. *megaterium* WSH002 (*B*. *m*. WSH002 hereafter), *i*MZ1055, has been previously reported^28^. This model included some obsolete annotations and was shown not to be very successful in predicting fluxes^29^. In this study, a GEM has been developed for *B*. *m*. DSM319. This model reconciles *i*MZ1055 and biochemical data specific to *B*. *m*. DSM319, taking into account other *Bacillus* metabolic models (including *i*Bsu1103 and *i*Bsu1147 for *B*. *subtilis*, and *i*WX1009 for *B*. *licheniformis*). Fig. 1 schematically represents the procedure of GEM reconstruction for *B*. *m*. DSM319. It should be emphasized that our model was able to accurately predict the metabolic functions and growth behavior of the strain. Model predictions were in agreement with the growth simulation results from Biolog phenotyping assays, as well as with several experimental data-sets reported in the literature.

## Results and Discussion

### Genome-scale reconstruction process

**Figure 2:**
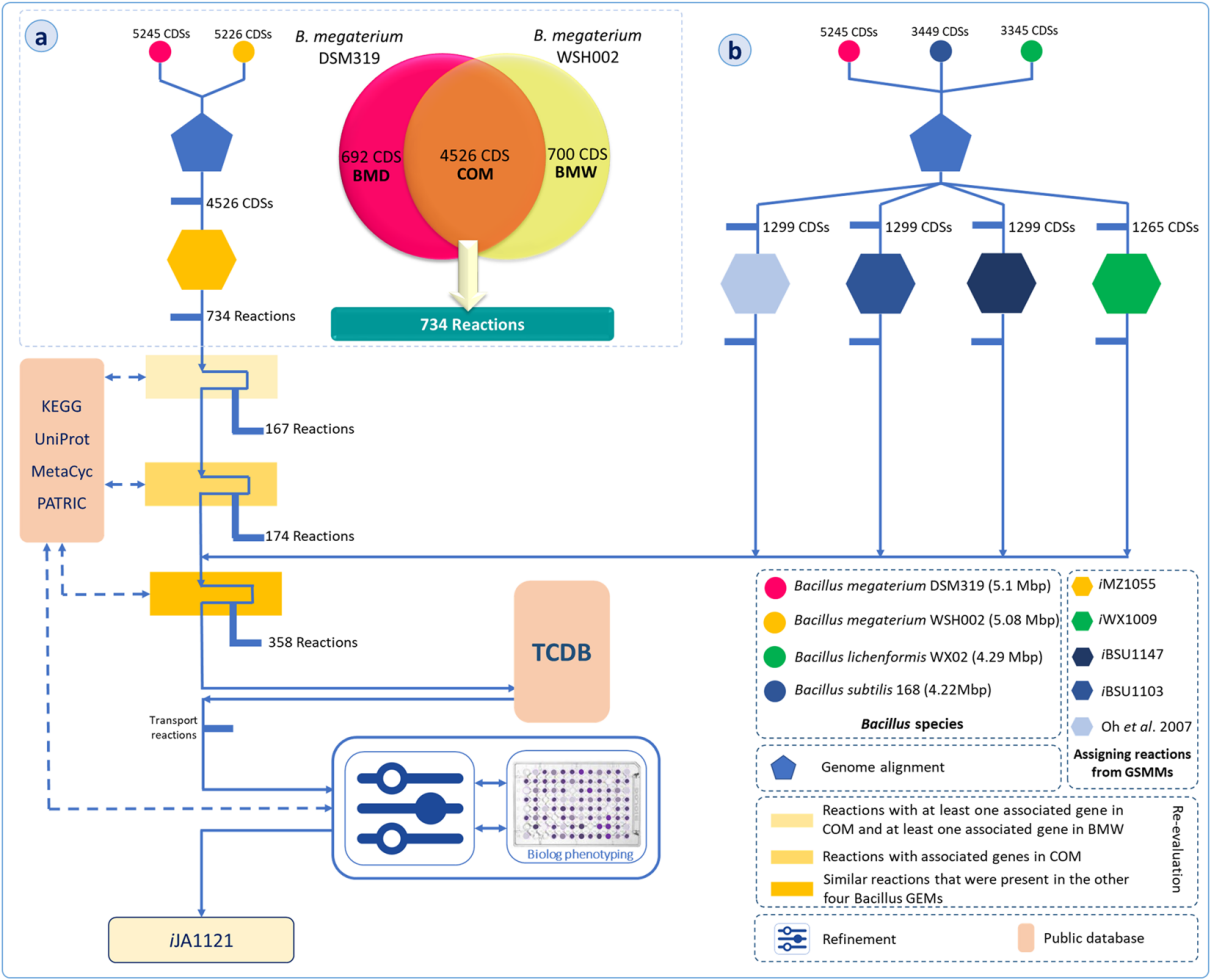
Schematic representation of the reconstruction process used for the genome-scale metabolic network of *Bacillus megaterium* DSM319. (**a**) Based on genome alignment, 734 reactions of *i*MZ1055 were included in the reconstruction of the initial draft network. (**b**) The initial draft network was modified by performing several manual curation steps, utilization of *Bacillus* species GenBank genomes for multiple-alignment and further addition of relevant reactions to the model, and refining the model based on phenotyping data. Solid arrows represent the process direction and dotted lines relate to the operations that were performed based on the available data in public databases. “167 Reactions”, “174 Reactions” and “358 Reactions” refer to “the reactions that had at least one associated gene in COM and at least one associated gene in BMW”, “the reactions that were associated with some gene(s) of COM” and “the similar reactions that were present in the other four Bacillus GEMs”, respectively (see Supplementary Information).

According to a reconciliation process, we used genome sequences of *Bacillus* species to identify potential reactions that should be present in the GEM of *B*. *m*. DSM319. Genome-wide multiple sequence alignment was performed prior to refinement and validation, in order to find orthologs. The detected homologous gene pairs were applied as references for identification of similar gene-protein-reaction (GPRs) associations to reconstruct the draft network. For this purpose, the homologous gene pairs of *B*. *m*. DSM319 and *B*. *m*. WSH002 were initially identified using Mauve^30^. Based on the results, 4526 coding sequence (CDS) homologous pairs were determined, which will be referred to as the “COM” genes (see Supplementary Information). Moreover, *B*. *m*. DSM319 had 692 CDSs with no obvious homolog in the *B*. *m*. WSH002 genome. This set of genes will be called “BMD” genes (Fig. 2a). Those genes which are present in *B*. *m*. WSH002, but not in the *B*. *m*. DSM319 genome, will be called “BMW” genes. Further information about the reconstruction process is presented in Supplementary Information.

In the next step, the draft network was manually curated in order to find potential errors. Altogether, we resolved 314 errors, including the modification of GPR associations, EC numbers, metabolites, addition of several complex enzymes and isozymes, as well as modification of the relationships among genes using Boolean logical operators.

In order to find any potential missing reactions which are present in the other four *Bacillus* GEMs, we carried out a genome-wide multiple sequence alignment for *B*. *megaterium*, *B*. *subtilis*, and *B*. *licheniformis*. Based on the results (Fig. 2), 358 reactions were added to the draft network. Furthermore, the BMD genes, which are present in *B*. *m*. DSM319 only, together with their associated reactions which were obtained by KEGG API^31^, were added to the draft network.

Finally, the manual refinement step in the reconstruction of the *B*. *m*. DSM319 network was performed by examining the inconsistencies between the *in silico* predictions and experimental results (Biolog phenotyping and literature). Figure 2b schematically represents the entire process of GEM reconstruction for *B*. *m*. DSM319.

### Phenotyping assays

**Figure 3:**
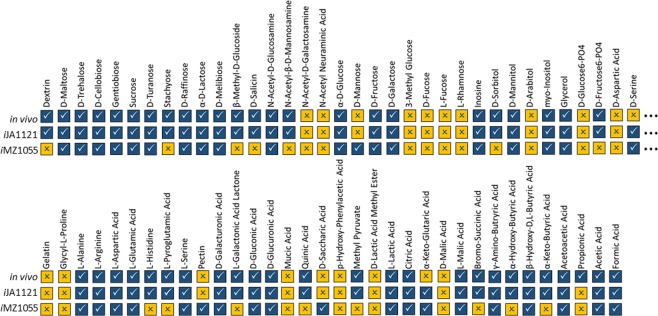
Results of *in silico* and phenotyping experiments based on growth on 69 different carbon sources (☑: Growth/True, ☒: No-Growth/False). The experimental results were obtained for *Bacillus megaterium* DSM319 based on the procedure given in the Materials and Methods.

We performed Biolog phenotyping experiments to examine the capability of the draft network to predict growth behavior on different carbon sources. *B*. *m*. DSM319 was found to be capable of growing on 49 out of 69 different carbon sources based on triplicate independent experiments (Fig. 3). Growth on different carbon sources was simulated, as explained in the Materials and Methods section, using FBA^32^. Further refinements of the draft network were performed based on the observed phenotyping results and *in silico* predictions. For every carbon source, an independent simulation was run. Among 69 different carbon sources, 60 carbon sources matched transport reactions while nine were known as intracellular metabolites, with no transport reaction in the draft model. Therefore, in order to determine potential membrane transporters for the 9 suspected intracellular metabolites, a literature review was performed seeking any reported gene-protein associations for the missing 9 membrane transporters. The result was used for homology searches in *B*. *m*. DSM319. Thus, we identified genes encoding uncharacterized membrane proteins responsible for transport of l-pyroglutamic acid (PA) and d-salicin metabolites, and added the relevant transport reactions to the model. For the other 7 intracellular metabolites, interim hypothetical transport reactions were correspondingly added to the model. A hypothetical transport reaction was preserved in the model only when simulating growth on the particular corresponding carbon source. The addition of those hypothetical transport reactions improved model accuracy in all 7 cases. Our results indicated that 56 out of 69 *in silico* predictions for growth on different carbon sources were compatible with phenotyping assays. Overall, 14 discrepancies were fixed by changing reaction reversibility or filling the gaps based on literature mining (see Table S1 in Supplementary Information). For example, *in silico* simulations were initially not able to correctly predict the capability of *B*. *m*. DSM319 to metabolize PA. In the draft network, PA had been considered as an intracellular metabolite. Our investigations showed that there was a need to add the pyroglutamase (ATP-hydrolyzing) reaction encoded by BMD_2469. Also, it was reported that the uncharacterized membrane proteins DUF969 and DUF979 are involved in PA transport in *B*. *subtilis*^33,34^. Based on the protein homology search, we decided to add a transport reaction and assumed that it is encoded by BMD_1100 and BMD_1101. The addition of these two reactions resulted in the model becoming capable of accurately predicting growth on PA as the sole carbon source. By refining and filling in the gaps, the GEM, which will be referred to as *i*JA1121, reached almost 96% accuracy in predicting substrate utilization (see Supplementary Information for the model in SBML format). It should be noted that *i*JA1121 was checked using Memote^35^.

By comparison, out of 69 different carbon sources, *i*MZ1055, the previous GEM for another strain of *B*. *megaterium*, predicted growth for only 38 carbon components, where 21 predictions were not consistent with the phenotyping experimental data. Overall, *i*MZ1055 could estimate growth for about 70% of carbon sources. Although this outcome could be attributed to metabolic differences between strains and their ability to grow on different carbon sources, it might in part be due to possible deficiencies within *i*MZ1055. A large number of non-growth/growth (*in silico*/*in vivo*) predictions (Fig. 3) demonstrates that some cellular functions were neglected in *i*MZ1055. It seems that there might exist other parallel metabolic pathways and isozymes that were not taken into account in *i*MZ1055.

### Constraint-based metabolic model of *B*. *m*. DSM319 and its performance in predicting metabolic behavior

The final GEM of *B*. *m*. DSM319, *i*JA1121, includes 1121 genes, 1349 metabolites, and 1709 reactions. Table 1 presents an overall comparison of GEMs available for *Bacillus* species. Compared to *i*MZ1055, *i*.*e*., the previous *B*. *megaterium* model, *i*JA1121 includes an increased number of metabolites, reactions, and genes.

**Table 1:** *Bacillus* species genome-scale metabolic models overview.

Model	*i*JA1121	*i*MZ1055	*i*Bsu1147	*i*Bsu1103	Oh *et al*., 2007	*i*WX1009
Species name	*B*. *m*. DSM319	*B*. *m*. WSH002	*B*. *s*. 168	*B*. *s*. 168	*B*. *s*. 168	*B*. *l*. WX02
Reactions	1709	1112	1742	1443	1020	1762
Metabolites	1349	993	1456	1145	988	1141
Genes	1121	1055	1147	1103	844	1009
Transport reactions	190	196	290	205	232	176
Refs.	This study	^28^	^58^	^59^	^70^	^60^

**Figure 4:**
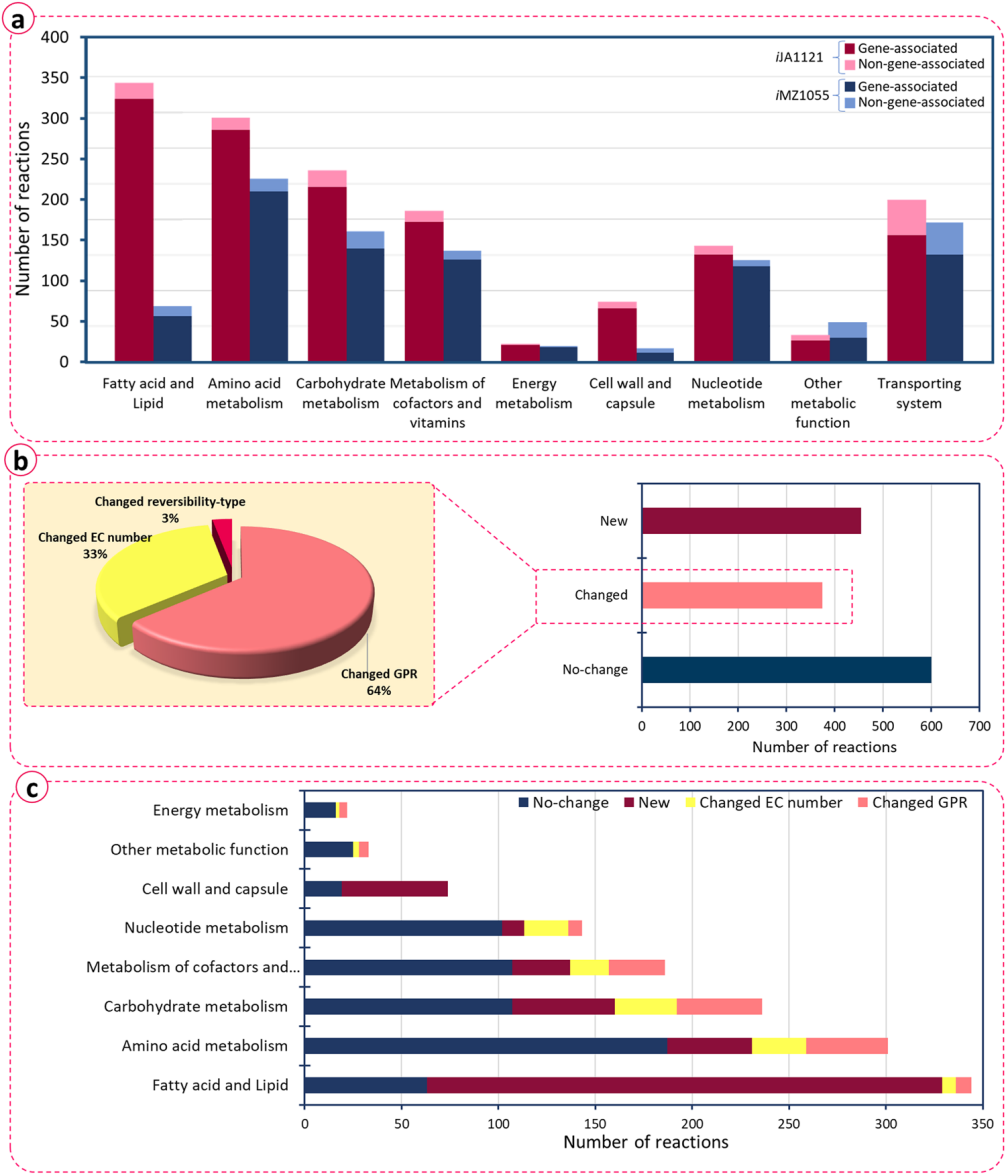
Features of *i*JA1121, (**a**) distribution of *i*JA1121 and *i*MZ1055 reactions in different subsystems, (**b**) classification of reactions in *i*JA1121 based on the modifications, (**c**) modifications made to the draft network ranked in different subsystems. “No-change” refers to those reactions that were added directly from iMZ1055 based on homology. “Changed” represents those reactions that were added from iMZ1055 based on homology, but with alterations in their GPR associations, EC numbers and/or reversibility-type. “New” refers to those reactions which were newly added during the reconstruction process.

We inspected the differences between *i*JA1121 and *i*MZ1055, in order to better understand the different metabolic capabilities of the two *B*. *megaterium* strains. The results of this study are summarized in Fig. 4. We classified the reactions into 7 categories based on their metabolic subsystems. In all the metabolic subsystems, the number of reactions in *i*JA1121 is larger than in *i*MZ1055. The largest difference is observed for the fatty acid and lipid subsystem, where the number of reactions is approximately six-fold higher in *i*JA1121. In *i*MZ1055, teichoic acids and fatty acids were presented as lumped reactions with lumped species. However, *i*JA1121 explicitly takes into account the reactions producing fatty acids and lipids. In most other metabolic subsystems, the situation was similar. Specifically, *i*JA1121 includes more reactions for cell wall and capsule synthesis, metabolism of amino acids, carbohydrates, cofactors, and vitamins. This considerable difference in the number of reactions prompted us to perform an in-depth investigation of the type of differences between the two models. All the reactions in *i*JA1121 were categorized into three classes, based on how they were added to the GEM (Fig. 4b): “No-change” refers to those reactions that remained unchanged, *i*.*e*., were added directly from *i*MZ1055 based on homology. “Changed” represents those reactions added from *i*MZ1055 based on homology, but with alterations in their GPR associations, EC numbers and/or reversibility-type. Finally, “New” refers to those reactions which were newly added during the reconstruction process. Accordingly, 42% of the reactions in *i*JA1121 are added directly (“No-change”), based on the information in biochemical databases and literature. Among the alterations that were made for the reactions in the “Changed” group that comprised 26% of the reactions, the changes of GPR associations were the most common. This is presumably due to the automatic annotation of genes by the modelSEED pipeline^28^. In addition, some of the EC numbers of *i*MZ1055 were obsolete, *i*.*e*. they were removed in the most recent release of the KEGG database. The other 32% of the reactions were those that were not included in *i*MZ1055. Fig. 4c shows how the changes are distributed over each of the metabolic subsystems. As expected, subsystems with more ambiguity in biochemical databases bore more changes^36^. From Fig. 4c, it can be observed that fatty acid and lipid, as well as cell wall and capsule subsystems have the highest ratio of added reactions. Also, amino acid metabolism, carbohydrate metabolism, metabolism of cofactor and vitamins, and nucleotide metabolism subsystems contained a large number of changes. These changes were based on the up-to-date information in biochemical databases and the literature data, and resulted in addition of new reactions and alterations of several EC numbers and GPR associations. On the other hand, fewest changes in terms of the newly added reactions occurred in the energy metabolism subsystem. In this subsystem, most changes were related to reaction parameters, including some EC numbers and some GPR associations.

### Prediction of growth for DSM319 and its derivatives

**Figure 5:**
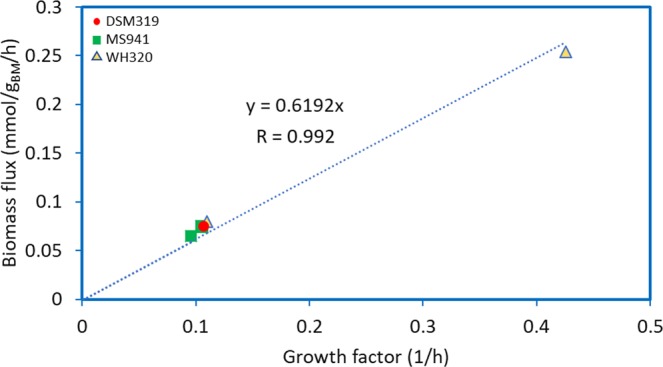
Results of growth simulations under different glucose uptake fluxes. The linear equation intercept is forced to zero (Pearson R = 0.99, *p*-value = 1.2e-05).

We characterized the growth behavior of *B*. *m*. DSM319, as well as that of its derivatives, namely *B*. *m*. MS941 (*∆nprM*), *B*. *m*. WH320 (*∆lacZ*) and *B*. *m*. WH323 (*XylA1-spoVG-lacZ*) (Table 2)^37^. All strains were cultivated in M9 medium with glucose as the sole carbon source, under aerobic chemostat conditions. Using FBA, the growth rates were calculated for the mentioned conditions. Mutations in the derivative strains were reported not have a remarkable influence on glucose-based growth^37,38^. To simulate the growth of *B*. *m*. DSM319 in the M9 medium, the lower boundary for all exchange reactions, except those related to the metabolites in the M9 medium, was set to zero. There was a significant correlation between the simulation results and the experimental data (Fig. 5); indicating an acceptable agreement between the two (Pearson R = 0.99, *p*-value = 1.2 × 10^−05^).

**Table 2:** Simulation conditions for different *B*. *m*. DSM319 strains.

Specific growth rate	Unit	DSM319	MS941		WH320	WH323
	*1/h*	0.106	0.11	0.426	0.096	0.107
Glucose uptake rate	*mmol/g*_*BM*_*/h*	1.52	1.62	5.17	1.31	1.53
Acetate uptake rate	*mmol/g*_*BM*_*/h*	0.15	0.17	0.6	0.17	0.16

Further investigation of the growth behavior of *B*. *megaterium* mutants was carried out by applying the results reported by Wang *et al*.^16^ to the analysis of MS941 and WH320 strains under high cell density conditions. To estimate glucose uptake fluxes and growth rates, Eqs. (1) and (2) were applied^39^.

1 $${v}_{glc,i}=\frac{{\mu }_{i}({C}_{glc,i+1}-{C}_{glc,i})}{{x}_{i}(\exp ({\mu }_{i}\times \Delta {t}_{i})-1)}$$

2 $${\mu }_{i}=\frac{\mathrm{ln}({x}_{i+1}/{x}_{i})}{\Delta {t}_{i}}$$

*v*_*glc*_, *μ*, C_*glc*_, *x* and Δ*t* are glucose uptake rate, specific growth rate, glucose concentration, biomass concentration and time period, respectively. Subscript *i* refers to the time step.

**Figure 6:**
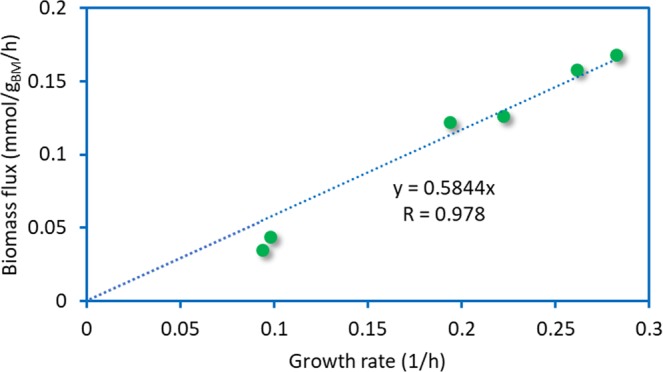
Comparison of biomass production flux obtained by FBA and experimental growth rate values. The linear equation intercept is forced to zero (Pearson R = 0.994, *p*-value = 1e-4).

We simulated these conditions by running FBA in the minimal medium and allowing glucose to enter the system at the flux obtained by Eq. (1). Simulations were performed under the assumption that both strains have similar growth behavior^16^. There was a good agreement between the biomass flux rates derived from simulations and the growth rate values (Fig. 6) (Pearson R = 0.994, *p*-value = 10^−04^).

### Simulation of *aroK* mutant and shikimic acid production

Limited availability and high price of precursors like shikimic acid and quinic acid is the main restraining factor for industrial production of essential aromatic compounds^40^. As a prominent compound in the pharmaceutical industry, shikimic acid is the main precursor for the synthesis of oseltamivir^14^. It is reported that a *∆aroK* mutant of *B*. *megaterium* can promote shikimate production^41^. The *aroK* gene encodes shikimate kinase, which catalyzes the bioconversion of shikimate to shikimate-3-phosphate in an ATP-dependent phosphorylation reaction.

**Figure 7:**
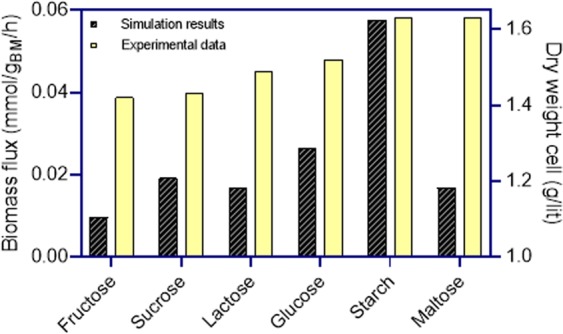
Comparison of biomass rate obtained by FBA and biomass dry cell experiments reported in the literature for different carbon sources including fructose, sucrose, lactose, glucose, starch, and maltose.

We simulated the *∆aroK* by setting the lower and upper bounds of the associated reaction to zero. Then, we analyzed the effect of different carbon sources on the growth behavior of the mutant strain by setting the lower bounds of the medium components and the carbon source to −1000 and −5 mmol/g_BM_/h, respectively. There was a significant correlation (R = 0.63, *p*-value < 0.05 in the Pearson correlation test) between biomass flux rate predictions by the GEM and the dry weight cell experiments (Fig. 7). When maltose was used as the sole carbon source, the experimental data and the simulation result were not in agreement. Excluding maltose from the results improved the correlation (R = 0.946, *p*-value < 0.05 in the Pearson correlation test). This observation suggests that the reactions for assimilation of maltose in the GEM need to be improved.

**Figure 8:**
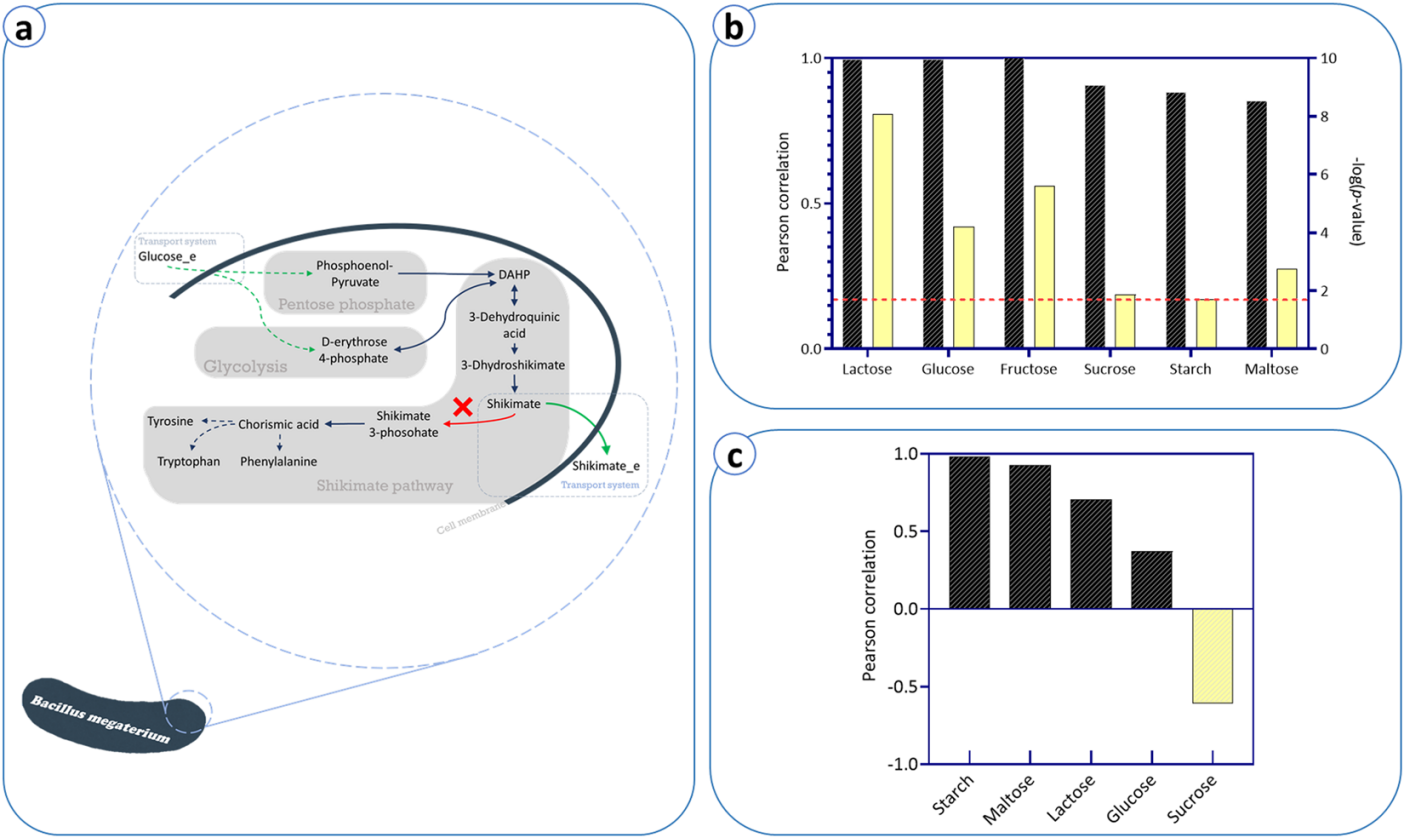
(**a**) Schematic of the shikimate production pathway. (**b**) Growth behavior analysis for *aroK* knock out mutant on different carbon sources. Pearson correlation coefficients and the -log(*p*-value) represent the results of the comparison study between *in silico* results and experimental data. The dashed line indicates the minimum −log(*p*-value) which was obtained for starch uptake. (**c**) Shikimate production simulations for *aroK* knock out mutant under different carbon sources.

Further simulations were performed to model the impact of different carbon sources on the growth rate of *∆aroK* strain (Fig. 8). By employing the experimental data reported in the literature^41^ and also applying Eqs. (1) and (2), we ran 43 FBA simulations under the aforementioned conditions. For each simulation, the lower bound of one carbon source was set based on the value obtained by Eq. (1). Then, FBA was performed to find the optimal growth rate. In the following step, we assumed the growth rate to be ≥90% of its maximum value (obtained in the former simulation), and another FBA was performed by taking the flux through shikimate dehydrogenase reaction as the objective function.

As demonstrated in Fig. 8b, this analysis yielded a significant Pearson correlation between predicted growth rate and the experimental cell dry weights. Pearson correlation coefficients for all substrates were higher than 0.85. Fructose, glucose, and lactose had the highest Pearson correlation coefficients. These findings confirm the ability of *i*JA1121 to predict mutant growth behavior.

We also investigated the potential of the model for predicting shikimic acid production by the *∆aroK* mutant. From Fig. 8c, one can observe that *in silico* shikimic acid production rates are reasonably consistent with the experimental data (for more information see Fig. S1 in Supplementary Information). For starch, maltose, and lactose, the highest Pearson correlation coefficients were observed, ranging from 0.98 to 0.71, while for glucose the smallest Pearson correlation coefficient, 0.37, was observed. However, the model failed to predict the shikimic acid production when sucrose was used as the sole carbon source. This can be attributed to different possible reasons. Sucrose has been reported to influence gene expression as a signal molecule in some microorganisms^42,43^. Also, differences in the metabolism of strains or possible errors in the reported experimental data could be other potential reasons for this discrepancy. These need further investigation.

### Formaldehyde inhibitory effect on growth

**Figure 9:**
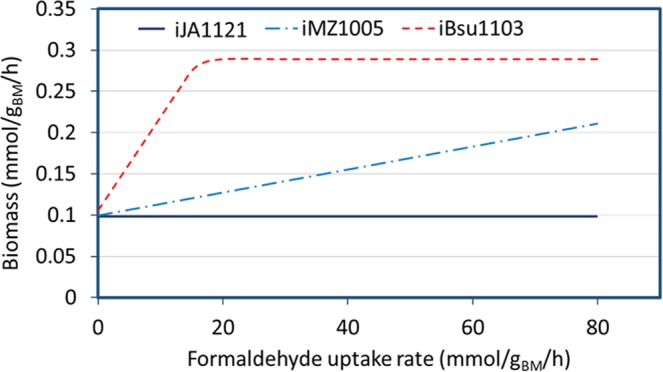
Results of formaldehyde sensitivity simulations in *B*. *megaterium* and *B*. *subtilis* using *i*JA1121, *i*MZ1055, and *i*BSU1103. For the simulations, formaldehyde flux was raised in a step-wise manner.

Formaldehyde (CH_2_O) is a prominent disinfectant that hinders microbial growth, particularly for *Bacillus* species, *e*.*g*., *B*. *megaterium* and *B*. *subtilis*^44^. It was shown that the GEMs generated for *Bacillus* species are incapable of predicting growth behavior under these conditions^29^. Herein, we simulated the glucose/formaldehyde co-metabolism using FBA for growth in minimal medium and glucose as the carbon source. For the formaldehyde uptake simulation, an exchange reaction was added to the model. In order to investigate the effect of formaldehyde addition, its flux was raised in a step-wise manner, and a number of FBAs were performed. The same simulations were run for the previous GEMs for *B*. *megaterium* (*i*MZ1055) and *B*. *subtilis* (*i*Bsu1103) starting from similar dilution rates. Fig. 9 depicts the result of formaldehyde metabolism analysis for *Bacillus* species. While *i*MZ1055 and *i*Bsu1103 show co-metabolism of glucose/formaldehyde as these GEMs contain pathways for assimilation of formaldehyde as a carbon source. However, *i*JA1121 was able to predict formaldehyde sensitivity in *B*. *megaterium*. From *i*JA1121 predictions, by increasing the formaldehyde uptake flux, the biomass production rate remained constant.

### Metabolic flux analysis using [U-^13^C] glucose

**Figure 10:**
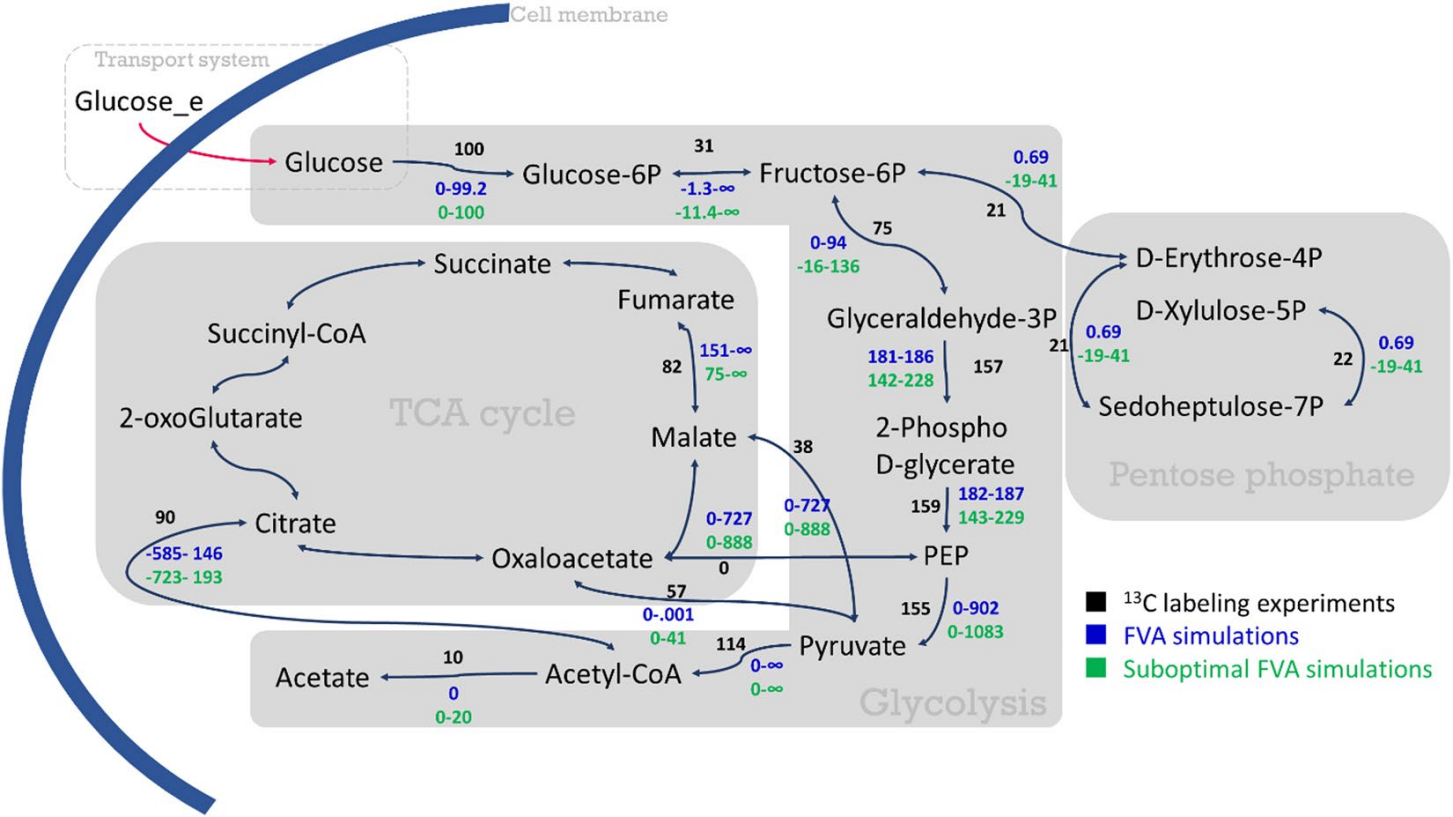
Comparison of FVA and suboptimal FVA simulations as well as ^13^C labeling experiments. Values in the figure are normalized with respect to the extracellular glucose uptake flux in percent. For a specific reaction, black values are the experimental ^13^C fluxes, blue values are the flux ranges obtained by FVA simulation and green values are intervals related to the suboptimal FVA simulation.

Results of FBA simulations typically do not reflect the exact metabolic behavior due to the existence of multiple optimal solutions^45^. In other words, alternate optimal solutions are independent flux distributions that optimize the objective function due to the existence of alternative biochemical pathways^46,47^. Using FVA (Flux Variability Analysis), one can predict the possible range of each flux under a certain optimal growth condition. Therefore, we ran four FVA simulations to investigate how the simulated fluxes agree with experimentally-measured ^13^C flux data^37^. The results are shown in Fig. 10.

In the simulations, it was assumed that glucose is taken up by *B*. *megaterium* directly, as isotopic measurements could not identify which glucose uptake pathway was active^47^. When comparing *in silico* results with experimental data, suboptimal FVA is more relevant, as it allows the objective function to be within an allowable range (*e*.*g*., ≥90% of maximum biomass production rate)^48^.Suboptimal FVA simulations were performed by setting the biomass producing reaction to 90% of the maximal value obtained by FBA. In Table 3 the reactions are listed. As can be seen in Fig. 10, predictions are in good agreement with experimental data and ^13^C fluxes generally fall within the intervals suggested by FVA.

**Table 3:** Metabolic reactions used to carry out the FVA simulations.

No.	Reaction	Does the experimental value fall within the predicted interval?
1	G6P[c] ↔ F6P[c]	Yes
2	ATP[c] + GLC[c] → H[c] + ADP[c] + G6P[c]	Yes
3	2PG[c] ↔ H2O[c] + PEP[c]	Yes
4	H[c] + ADP[c] + PEP[c] → PYR[c] + ATP[c]	Yes
5	E4P[c] + XUL5P[c] ↔ T3P1[c] + F6P[c]	Yes
6	T3P1[c] + S7P[c] ↔ E4P[c] + F6P[c]	Yes
7	2PG[c] ↔ 3PG[c]	Yes
8	FDP[c] ↔ T3P2[c] + T3P1[c]	Yes
9	XUL5P[c] + R5P[c] ↔ T3P1[c] + S7P[c]	Yes
10	2 OFER[c] + PYR[c] + COA[c] → 2 RFER[c] + ACCOA[c] + CO2[c] + 2 H[c]	Yes
11	ATP[c] + AC[c] + COA[c] ↔ ADP[c] + ACCOA[c] + PI[c]	Yes
12	PYR[c] + ATP[c] + HCO3[c] → H[c] + OA[c] + ADP[c] + PI[c]	No
13	H2O[c] + OA[c] + ACCOA[c] ↔ H[c] + COA[c] + CIT[c]	Yes
14	H2O[c] + FUM[c] ↔ MAL[c]	Yes
15	NAD[c] + MAL[c] → PYR[c] + NADH[c] + CO2[c]	Yes
16	ATP[c] + OA[c] → CO2[c] + ADP[c] + PEP[c]	Yes

There are several possible glucose assimilation mechanisms defined in the model. The predicted interval for the conversion of glucose to glucose-6-phosphate which is limited to [0, 100] confirms the existence of alternative reactions for assimilation of glucose. In the predictions, there are some intervals where their maximal or minimal values are found to be unbound. This pertains, for example, to the conversion of glucose-6-phosphate to fructose-6-phosphate, PEP to pyruvate, pyruvate to acetyl-CoA, pyruvate to malate to oxaloacetate. The appearance of these conditions is presumably related to the existence of futile cycles that are often inevitable in reconstructing a GEM. Such futile cycles linked by malate dehydrogenase, pyruvate carboxylase, and the malic enzyme were investigated experimentally^37^. The occurrence of futile cycles enhances the versatility of the organism to survive in a changing environment. Futile cycles could act as alternative pathways in order to regulate the cellular metabolism of the organism to function optimally^49^. Overall, there is an acceptable agreement between the *in silico* results and the ^13^C labeling experiments (for more information see Fig. S2 in Supplementary Information).

### Effect of carbon sources on fatty acid biosynthesis and lipid accumulation in *B*. *megaterium*

**Figure 11:**
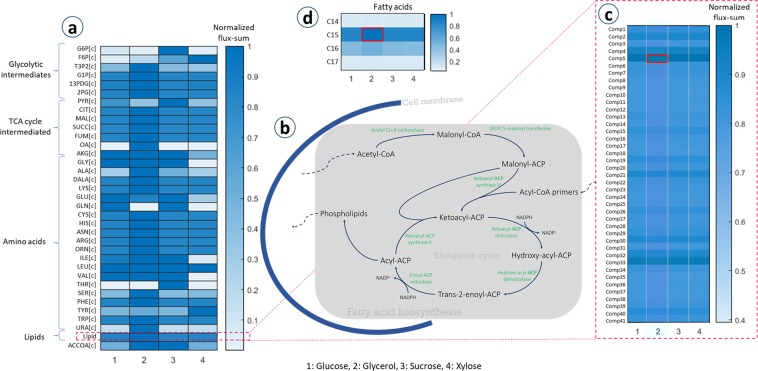
The heatmap representing the results of the flux-sum values of different components including glycolytic intermediates, TCA cycle intermediates, amino acids, and lipids. The comparisons were conducted for different carbon sources. (**b**) Schematic of fatty acid biosynthesis pathway. (**c**) Results of flux-sum values of the lipids constituting biomass. (**d**) Results of flux-sum values of the lipids constituting biomass.

Acetyl-CoA as the precursor of fatty acids plays an important role in the metabolic network (see Fig. 11b). In cellular metabolism, a fraction of pyruvate is converted to acetyl-CoA, which is the main precursor for fatty acids and biosynthesis of lipids^50^. Acetyl-CoA carboxylase catalyzes the conversion of acetyl-CoA to malonyl-CoA. The next metabolic step, transfer of malonyl-CoA to malonyl-[acyl-carrier-protein (ACP)] is catalyzed by [ACP]-S-malonyl transferase, and this initiates the synthesis of fatty acids. Then, malonyl-ACP reacts either with an acetyl-CoA for imitation, or with acyl-CoA primers for elongation, resulting in ketoacyl-ACPs, leading to a process known as the elongation cycle. The elongation cycle includes two NADPH-dependent reduction steps accompanying by a dehydratase reaction^51^.

We investigated the effect of using different carbon sources on fatty acid biosynthesis and lipid accumulation by comparing turnover rates of metabolites in the metabolic network. To do so, a flux-sum analysis was performed^52^. We compared the flux-sum values of metabolites in the network and the results are illustrated in Fig. 11. Four commonly used carbon sources reported in the literature (glucose, glycerol, sucrose, and xylose), were selected to run the simulations. The uptake flux rate of each carbon source was limited to 1 (mmol carbon)/g_BM_/h as the basis. Then, the flux-sum of the metabolites was calculated, as explained in the Materials and Methods section. Fig. 11a illustrates the flux-sum results for the metabolites, normalized with the maximum value of each metabolite. Although most of the metabolites show similar levels for different carbon sources, glycerol has a higher flux-sum distribution and xylose represents the lowest flux-sum values for most of the compounds. The higher the utilization of central metabolism, the higher the production of the biomass precursors. Glycerol with high flux-sum values for amino acids and glycolytic intermediates could potentially be a favorite source for fermentation products such as poly-hydroxybutyrate^53^ and fatty acids^54,55^. High turnover rates of amino acids known to be the sources of acyl-CoAs (leucine, isoleucine, and valine) indicate active biosynthesis of fatty acids^56^. Besides, growth on glycerol exhibited the highest flux-sum value for acetyl-CoA, which in turn favors lipid accumulation. These findings prompted us to compare the flux-sum values of lipids (Fig. 11c,d). The maximum flux-sum values of lipids and fatty acid intermediates (specified with red boxes in the figures) were applied as the references for normalization. As shown in Fig. 11c, the flux-sum distribution indicates that lipids have comparable flux-sums. It also shows that using glycerol as the carbon source leads to the highest flux-sum values for lipids. In addition, flux-sum values of fatty acids when glycerol is set as the carbon source are maximal (Fig. 11d). Among different types of fatty acids, C15-fatty acid had the highest, while C14- and C17-fatty acids had the lowest flux-sum values. These findings are consistent with the results reported by Scandella and Kornberg^57^ obtained during log-phase growth.

## Materials and Methods

### Genome files and genome-scale metabolic models

In order to identify the homologous gene pairs, GenBank genome (.gbk) files for *B*. *m*. DSM319, *B*. *m* WSH002, *B*. *subtilis* 168 (*B*. *s*. 168) and *B*. *licheniformis* WX02 (*B*. *l*. WX02) containing genomic sequences and annotations were downloaded (with accession numbers CP001982.1, CP003017.1, CP010052.1, and AHIF00000000.1, respectively). Four GEMs, *i*MZ1055 for *B*. *m* WSH002^28^, *i*Bsu1147^58^ and *i*Bsu1103^59^ for *B*. *subtilis* 168 and *i*WX1009 for *B*. *licheniformis*. WX02^60^ were obtained for comparison and/or were used as the reconstruction templates.

### Genome-scale metabolic network reconstruction

As the first step, a homology search was done based on progressive multiple alignments using Mauve version 2.4.0, using its default parameter settings. Mauve is a computational framework for multiple genome alignments in the presence of large-scale evolutionary events^30^. Accordingly, the related GenBank genome files containing genomic sequences and annotations were downloaded to identify gene orthologous pairs using the ‘progressiveMauve’ tool. Then, based on the results gained by Mauve, the associated reactions in *i*MZ1055 were identified and used as the basis of the draft network. In the second step, the refinement of the draft network was automatically performed (and then manually curated) based on public biochemical databases such as KEGG^61^, MetaCyc^62^, UniProt^63^, and PATRIC^64^. In the next step, with a similar strategy, we found all of the relevant genes and reactions which were present in *i*Bsu1103^59^, *i*Bsu1147^58^ and *i*WX1009^60^ and their corresponding GenBank genome files. Then, according to the information on TCDB^65^, the transport equations were added to the draft network. Finally, the gaps of the model were identified and corrected, based on the phenotyping results as well as the experimental data reported in the literature.

### Flux balance analysis and biomass objective function

FBA is one of the popular methods for analyzing the flux of reactions in metabolic networks under steady-state conditions^66,67^. FBA assumes that no accumulation of metabolites occurs during growth (*dc*/*dt* = 0), while a cellular objective, typically biomass production rate, is optimized. Based on the FBA, a linear programming problem is defined as follows:

$${\rm{maximize}}:Z=\sum _{j}{c}_{j}{v}_{j}$$

$${\rm{subject}}\,{\rm{to}}:\sum _{j}{S}_{ij}{v}_{j}=0$$

$${v}_{j}^{\max }\le {v}_{j}\le {v}_{j}^{\max }$$

where *Z* is a linear function related to the cellular objective and the coefficients *c*_*j*_ determine the weights of the reaction *j*. Also, *v*_*j*_ refers to the flux rate of the reaction *j* in the network and *S*_*ij*_ is the stoichiometric coefficient of metabolite *i* in reaction *j*. In this approach, each flux, *v*_*i*,_ is constrained to the given minimum and maximum values. To perform FBA, a biomass producing reaction, representing a weighted ratio of components in cell dry weight, is maximized based on the evolutionary assumption^68,69^. In this study, the biomass producing reaction of the *i*MZ1055 model is used, which in turn is based on the biomass composition of the *B*. *subtilis* model^70^. Unless stated otherwise, the biomass production rate was used as the objective function in FBA simulations.

### Flux variability analysis

FVA is a mathematical tool for finding the minimum and maximum possible fluxes of each reaction, while other constraints are satisfied and the objective function takes the optimal (or, suboptimal) value^71^. In this study, suboptimal FVA was used to find the minimum and maximum possible fluxes of each reaction while the objective function was bound to 90% of its maximal value (achieved by FBA).

### Flux-sum analysis

In order to illuminate the role of metabolite in the network and study turnover rates of the metabolites, we used flux-sum analysis^72^. The flux-sum of metabolite *i* is defined as the summation of all consumption or generation fluxes as follows^52^:

$${\phi }_{i}=\frac{1}{2}\sum _{j}|{S}_{ij}{v}_{j}|$$

The existence of possible alternate optimal solutions can result in ambiguity in the interpretation of FBA solutions. Thus, to identify the most plausible flux distribution, the minimization of the sum of total fluxes makes the calculation ${\phi }_{i}$ feasible^73^.

### Experimental methods

To discern the phenotypic pattern of *B*. *m*. DSM319, the ability of the strain to grow on different carbon sources was tested using the Biolog GEN III microplate. This was used to analyze the strain under 94 phenotyping tests. As specified by the manufacturer, a pure culture of the strain was incubated at 30 °C overnight on an agar plate with 5% sheep blood, and then suspended in a special inoculating fluid at the predetermined cell density (90–98% Transmittance). Then, 100 ml of the cell suspension was inoculated into each well of the GEN III MicroPlate™. The microplate was incubated at 30 °C for 24 hours. Readouts of growth-dependent color change were obtained using a microplate reader and interpreted based on the Biolog protocols.

## Conclusion

Aerobic Gram-positive *Bacillus* species are commonly used in biotechnology, especially in food, pharmaceutical, and environmental processes. Lack of knowledge about growth behavior of these organisms complicates the design and monitoring of industrial microbial processes. Generating constraint-based models that can predict growth behavior is an important step in addressing this knowledge gap. In this regard, several GEMs for *Bacillus* species have been constructed. After the reconstruction of the GEM for *B*. *subtilis* 168, generated by Oh *et al*., *in silico* models were developed for *B*. *m*. WSH002 (*i*MZ1055) and *B*. *l*. WX02 (*i*WX1009). In addition, two other GEMs for *B*. *subtilis* 168, *i*Bsu1103 and *i*Bsu1147, were published more recently. It has been reported that existing genome-scale models for *B*. *megaterium* could not correctly predict the utilization of amino acids and some carbon sources^29^. Herein, we present a GEM for *B*. *m*. DSM319, *i*JA1121. The model includes 1709 reactions, 1349 metabolites with 1121 genes. Barring exchange reactions, 91% of the reactions are gene-associated.

Following an automated approach, the draft network was curated manually by updating the GPR associations and EC numbers as well as adding several reactions. The genome-scale model was validated using our own experimental data on Biolog phenotyping and published data on *B*. *m*. DSM319 growth on different carbon sources (available on PubMed). FBA and FVA were applied to perform the simulations. Our findings suggested a better agreement of *in silico* predictions and experimental data for *i*JA1121. Results of carbon source utilization experiments and the model predictions matched in 96% cases. Moreover, the growth behavior of different mutant strains of *B*. *m*. DSM319 was studied and results indicated a very good match between *i*JA1121 predictions and *in vivo* data, with very few exceptions. For instance, the simulation of shikimate production in *aroK* knock out worked well on all tested carbon sources except sucrose. Further investigations were performed by comparing *in silico* results with ^13^C labeling experiments. Regarding suboptimal FVA results, the experimental ^13^C fluxes fell within the predicted intervals. In conclusion, *i*JA1121 seems to offer a clear improvement over *i*MZ1055, where the success rate of the previous model in accurately predicting growth behavior in the same type of simulations was only 70%.

## Supplementary information

Supplementary Information

iJA1121

Dataset 1
