# Supplementary material for: Manually curated genome-scale reconstruction of the metabolic network of Bacillus megaterium DSM319
Source: Sci Rep. 2019 Dec 10;9:18762. doi: 10.1038/s41598-019-55041-w (PMC6904757; doi:10.1038/s41598-019-55041-w)
Supplement: Supplementary file 1 — Supplementary Information [file 41598_2019_55041_MOESM1_ESM.pdf]

Supplementary Information

Manually curated genome-scale reconstruction of the metabolic network of *Bacillus megaterium* DSM319

Javad Aminian-Dehkordi[1], Seyyed Mohammad Mousavi[1,*], Arezou Jafari[2], Ivan Mijakovic[3,4], Sayed-Amir Marashi[5,*]

[1]Biotechnology Group, Department of Chemical Engineering, Tarbiat Modares University, Tehran, Iran

[2]Department of Chemical Engineering, Tarbiat Modares University, Tehran, Iran

[3]Department of Biology and Biological Engineering, Chalmers University of Technology, Göteborg, Sweden

[4]Novo Nordisk Foundation Center for Biosustainability, Technical University of Denmark, Denmark

[5]Department of Biotechnology, College of Science, University of Tehran, Tehran, Iran

[*] *Corresponding Authors*: mousavi_m@modares.ac.ir, marashi@ut.ac.ir

The reconstruction process used for the genome-scale metabolic network of *Bacillus megaterium* DSM319

*i*MZ1055 contains 43 genes which are marked as “new annotated genes”. Among these, 27 genes belong to COM genes. The remaining 16 genes, were incorrectly associated with reactions. However, for each of these reactions, we were able to find other genes in *B. m.* DSM319 genome with the same E.C. numbers. The corrected gene-reaction associations were added to the draft network.

In the next step, we identified those metabolites and reactions of *B. m.* WSH002 which are present in *i*MZ1055. Overall, 734 non-transport reactions of the *B. m.* WSH002 network were taken into account. Among them, 567 reactions were associated with some COM gene(s), and none with BMW genes. These reactions were assumed to be present in *B. m.* DSM319 metabolism, after manually removing the incorrect reactions. The presence of all these reactions in *B. m.* DSM319 metabolism was confirmed using KEGG, MetaCyc and UniProt databases and also by modifying 174 reactions. The other 167 reactions had at least one associated gene in COM and at least one associated gene in BMW. We reevaluated these reactions manually. In addition, reactions in *i*MZ1055 which were found to be (potentially) associated with some BMW gene were inspected manually, in order to identify any other possible reaction of *i*MZ1055 not included in our draft network. In this step, 12 more genes with 9 associated reactions were added to the draft model. Finally, all non-gene-associated reactions of *i*MZ1055 were scrutinized to ascertain their correctness, regarding the available data in the literature, as well as KEGG, UniProt and MetaCyc databases. In this step, we added 61 non-gene-associated reactions to the draft network.

Gap filling based on the phenotyping assays

Table S1- Simulation of growth under different carbon sources that were not predicted correctly by the model initially. Proper actions were made based on the information on databases and literature mining. Descriptions are presented in the Table.

Number	Carbon sources	Experiment	Simulation result before gap filling	Simulation result after gap filling	Action(s)	Description
1	β-Methyl-D-glucoside	+	-	+	Adding a new reaction	Based on the information on MetaNetX, BMD_1886 and BMD_2126 that encode EC number 3.2.1.23 were identified.
					Adding a new reaction	Based on the information on MetaNetX, BMD_1850 that encodes EC number 5.1.3.3 was identified.
2	D-salicin	+	-	+	Adding a new reaction	Based on the information on KEGG, BMD_3359, BMD_1020 and BMD_0773 that encode EC number 3.2.1.86 were identified.
					Adding a transport reaction	Based on the homology search, BMD_1283 was identified (homology: ptsH, BSU13900).
3	D-sorbitol	+	-	+	Adding a new reaction	The reaction grxn1 was added based on the information on KEGG database: BMD_3588 (1.1.1.14)
4	D-glucuronate	+	-	+	Adding a transport reaction	A hypothetical transport reaction was added (Ref.: *i*BSU1103, *i*BSU1147, *i*MZ1055)
5	N-acetyl-β-D-mannosamine	+	-	+	Adding a transport reaction	A hypothetical transport reaction was added (Ref.: *i*BSU1103, *i*BSU1147)
6	L-pyroglutamic acid	+	-	+	Adding a new reaction	Based on the information on KEGG, BMD_2469 or BMD_0962 that encode EC number 3.5.2.9 were identified.
					Adding a transport reaction	Based on homology search, BMD_1101 and BMD_1101 were identified (homology search: 28830929, 1106127).
7	Acetic acid	+	-	+	Change reversibility-type	The reaction RxnBME0373 was reversible (Ref.: *i*MZ1055)
8	Formic acid	+	-	+	Adding a new reaction	Based on homology search, BMD_3473 was identified.
9	Methyl pyruvate	+	-	+	Adding a transport reaction	A hypothetical transport reaction was added.
11	β-hydroxy-D,L butyric acid	+	-	+	Adding a transport reaction	A hypothetical transport reaction was added.

Table S1- Continued

Number	Carbon sources	Experiment	Simulation result before gap filling	Simulation result after gap filling	Action(s)	Description
12	Propionic acid	+	-	+	Adding a transport reaction	A hypothetical transport reaction was added.
13	Quinic acid	+	-	+	Adding a new reaction	The reaction grxn1 was added based on the information on the KEGG database.
					Change reversibility-type	The reaction RxnBME0292 was reversible (Ref.: *i*BSU1147, *i*BSU1103)
14	Fructose 6-phosphate	+	-	+	Adding a transport reaction	The reaction Tr5 was added (Ref.: iYO844: F6Pt6_2, *i*BSU1103: Fructose 6-phosphate transport via phosphate antiport)

Details of *in silico* simulations

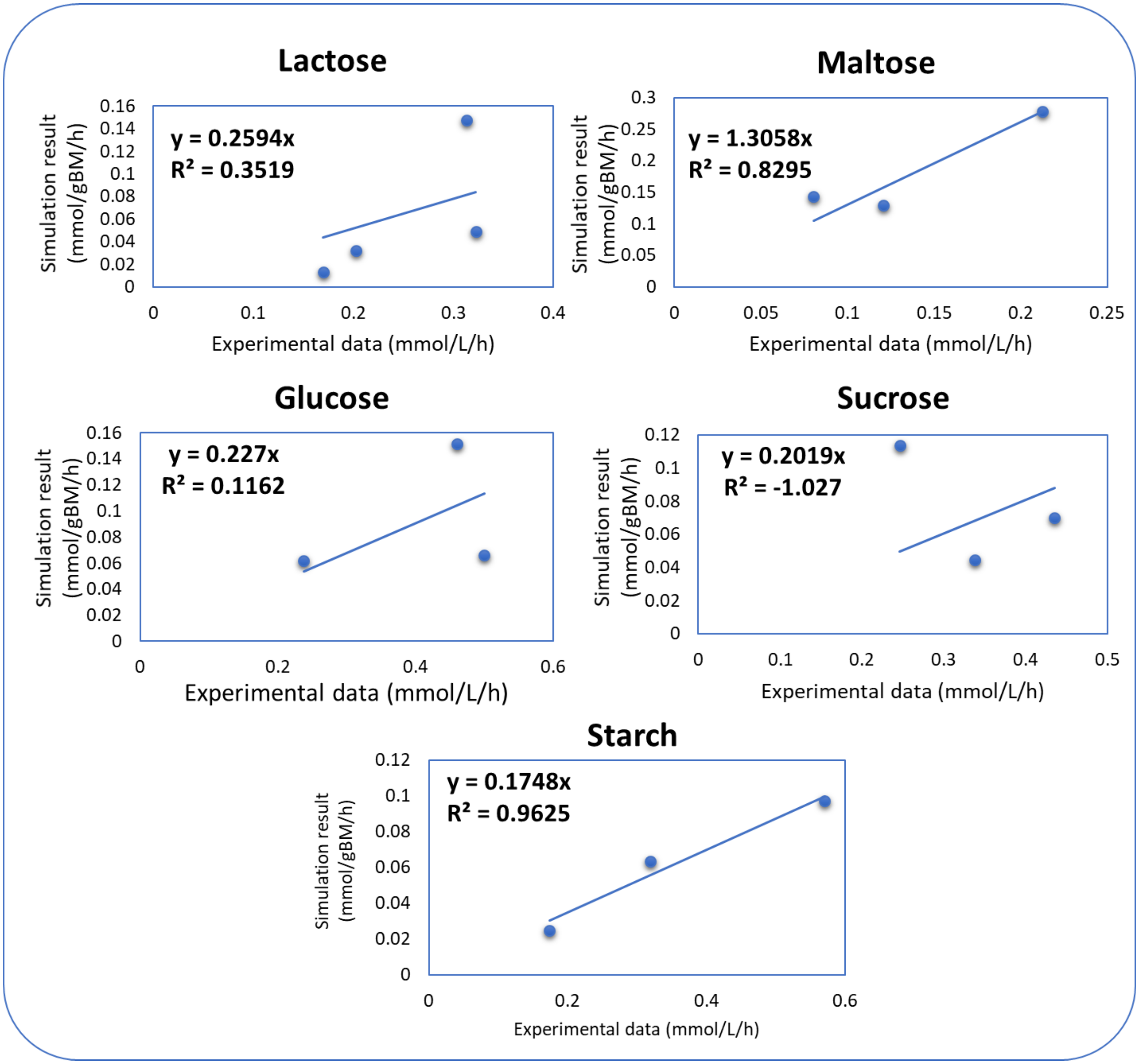

Figure S1- Shikimate production simulations for *aroK* knock out mutant under different carbon sources. In all figures, the linear equation intercepts are forced to zero

Details of *in silico* simulations

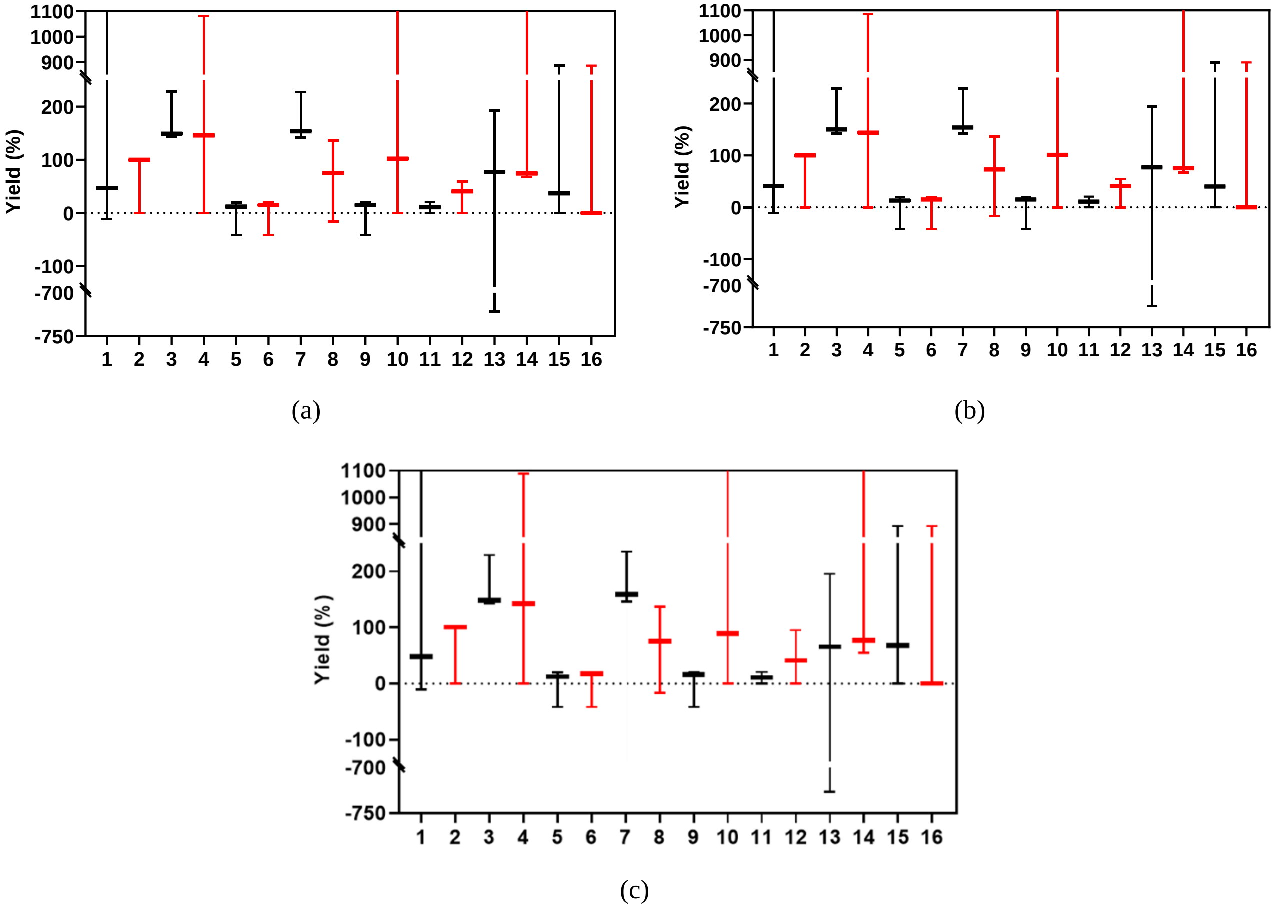

Figure S2- Results of suboptimal FVA simulations under different dilution rates in comparison to the ^{13}C labeling experiments, (a) v_{glc} = 1.47 mmol/g_{BM}/h, (b) v_{glc} = 1.62 mmol/g_{BM}/h, and (c) v_{glc} = 5.17 mmol/g_{BM}/h.
